# The impact of influencer marketing in the tourism industry: A digital marketing perspective

**DOI:** 10.1371/journal.pone.0338423

**Published:** 2025-12-12

**Authors:** Md. Asaduzzaman Babu, Hafsa Akter Urmi, Abzal Hosen Tofayel, Md. Mehedul Islam Sabuj, Md Tamjid Ul Alam

**Affiliations:** 1 Department of Marketing, Hajee Mohammad Danesh Science and Technology University, Dinajpur, Bangladesh; 2 Faculty of Management, Multimedia University, Cyberjaya-, Malaysia; University of Buraimi, YEMEN

## Abstract

This study aims to examine how influencer marketing shapes tourists’ perceptions and purchase intentions in the tourism sector of Bangladesh. Specifically, it examines the impact of word of mouth, content characteristics, consumer trust, emotional connection, and brand awareness on consumer perception, as well as how these perceptions influence purchase intention within a digital marketing context. A quantitative, cross-sectional design was employed, utilizing a structured online questionnaire distributed to active social media users who follow travel influencers. Data were collected from 400 respondents through non-probability purposive sampling, representing individuals familiar with influencer-generated travel content. The data were analyzed using Partial Least Squares Structural Equation Modeling (PLS-SEM) to test the proposed hypotheses and evaluate both measurement and structural models. Results revealed that word of mouth (β = 0.269, p < 0.001), content characteristics (β = 0.152, p < 0.05), consumer trust (β = 0.207, p < 0.05), emotional connection (β = 0.170, p < 0.05), and brand awareness (β = 0.200, p < 0.001) significantly influence consumer perception. In turn, consumer perception strongly predicts purchase intention (β = 0.561, p < 0.001). All five influencer-related factors; word of mouth, content characteristics, trust, emotional connection, and brand awareness significantly influence consumer perception, which in turn strongly predicts purchase intention. Among these, trust, word of mouth, and content quality were found to be the most influential predictors of success. The findings confirm that credible and engaging influencer communication enhances tourists’ perceptions and motivates travel-related purchasing behavior. The study provides actionable insights for tourism marketers and policymakers, emphasizing the importance of authenticity, message quality, and trust-building in influencer collaborations. It also highlights the need for integrating influencer campaigns into broader destination branding strategies. This research contributes theoretically by integrating Social Influence Theory and Source Credibility Theory into a single model. It provides new empirical evidence from an emerging tourism market (Bangladesh), expanding cross-cultural understanding of influencer marketing’s role in shaping consumer behavior in digital tourism.

## 1. Introduction

The phenomenon of influencer marketing, which utilizes social media influencers for promotional purposes, has enhanced tourism promotion. Traditionally, this effort was carried out in partnership with Destination Management Organizations (DMOs) to increase the attractiveness of destinations and visitor participation [1]. The stories and imagery are persuasive because travel influencers share powerful narratives about destinations; these ideals turn into highly influential, peer-like recommendations that resonate with contemporary travelers. Novel empirical research [2] on the ‘travel influencer construct’ demonstrates that trust is associated with authenticity, attractiveness, inspiration, and content quality. These four dimensions combine to amplify persuasive influence in a tourism-specific environment. From a consumer psychology standpoint, the role of travel influencers is considerable in the upper levels of the decision-making process of tourists, as they increase destination awareness, create attitudes, and enhance involvement through purchase behavior [3]. In addition, according to systematic reviews, influencer marketing has had a significantly higher effect on tourism since 2020 [4]. These effects are positively correlated with destination image, travel intention, and planning behavior. Building on the discussion, recent comparative research has distinguished between human and virtual influencers, viewing the former as distinct entities, where the latter may not be the optimal option for promoting cultural destinations.

Additionally, human influencers are more effective than Google Trends for promoting natural tourism atmospheres [5]. These variations demonstrate the importance of aligning influencer type and destination category in strategic marketing efforts. Another study also offers insight into sensitivity toward message truthfulness, revealing that travel influencers who tell personal stories and maintain consistent narratives positively contribute to credibility in their accounts. This relationship has been identified as an important antecedent of consumers’ responses [6]. These academic considerations suggest that influencer marketing in tourism sits at the intersection of storytelling, authenticity, and consumer engagement, presenting an area for research and a space for intervention.

The adoption and effectiveness of influencer marketing in the tourism industry, particularly in emerging digital economies, have not been well studied, in contrast to areas such as fashion [7]; Le [8]), beauty [9,4], and retail [10–12], where it has been thoroughly researched. Much of the prior literature has focused on well-established digital ecosystems with high levels of consumer-brand trust [13], where social media influencers have a significant impact on consumers’ attitudes and purchasing intentions [14,15]. This has led to a lack of understanding of how these mechanisms work in developing countries like Bangladesh, where the use of social media is still in its early stages and people’s trust in online sources is still building [16,17]. In addition, rather than viewing influencer marketing as a framework that alters consumer perception and buying intention, current research often treats influencer marketing constructions as separate entities. These factors include word of mouth, content quality, trust, emotional connection, and brand awareness. Research on the combined effects of these factors on digital influencers’ ability to sway tourists’ decisions is lacking. The cultural and contextual intricacies that influence consumer perception in collectivist countries, where social endorsement, reliability, and trust are more important than emotional appeal, are often overlooked by prior models. Hence, the study arises the following research question RQ1: How does influencer marketing affect consumer perception and purchase intention in the tourism industry of Bangladesh? RQ2: How does the digital environment influence influencer marketing constructs on consumer perception and purchase intention in the Bangladeshi context? Therefore, this study fills these gaps by developing and testing an integrated model that examines how influencer-driven factors affect consumer perception and purchase intention in the Bangladeshi tourism industry. This framework expands influencer marketing theory into a new sector and culture.

The tourism industry is shifting its marketing activities from traditional advertisements to digital-focused strategies and is increasingly relying on social media influencers to attract the attention of prospective travelers [1]. Although influencer marketing is a well-established practice in most industries, its applicability and effects in the tourism domain remain under-researched empirically [18]. A cascade of empirical research has demonstrated that influencer characteristics, including credibility, authenticity, and content quality, can have a significant impact on DI (destination image) and TI (travel intention). Nonetheless, their interaction is not well established in an overall digital marketing context [2]. In addition, prior studies have commonly identified and investigated the antecedents of influencer factors separately, rather than amalgamating various constructs, including word of mouth, content characteristics, trust, emotional connection, and brand knowledge, in a single model [3]. Interacting in such a fragmented manner does not necessarily provide a clear picture of how these factors integrate to affect tourists’ perceptions. Engaging in such a disjointed manner does not inherently provide a coherent understanding of how various elements converge to influence visitors’ perceptions. Consumer perception has been recognized as a significant intermediary between marketing messages and purchase intentions, although it has not been investigated within the framework of influencer-led tourist promotion [6].

By integrating several influencer-related criteria into a unified framework, this study enhances our understanding of how these aspects impact consumer perception and purchase intention. These factors include word of mouth, content quality, trust, emotional connection, and brand awareness. Additionally, it addresses a critical concern for managers: the potential of influencer marketing to generate interest in and trust in the products of tourist marketers in developing nations. Digital strategists, destination marketing groups, and tour operators can all benefit from the study’s findings by gaining a deeper understanding of how to select influencers, create high-quality content, and effectively communicate with their target audiences. This study examines influencer marketing concepts in a developing economy tourism context, contributing to the growing body of research on digital marketing and consumer behavior. Additionally, it contributes to global discussions regarding cultural differences in product responses by demonstrating that in collectivist nations, trust, authenticity, and content relevance, rather than merely emotional attachment, influence consumers’ perceptions and purchasing decisions. The study has important theoretical implications for broadening the scope of influencer marketing beyond traditional industries, as well as practical implications for guiding the strategic development of tourism marketing campaigns in Bangladesh and similar countries undergoing digital transformation.

## 2. Literature review

### 2.1 Theoretical background

According to the Social Influence Theory, proposed by Kelman [19] and Cialdini [20], people’s opinions and actions are influenced by the ideas, actions, and proposals of those around them in their social environment. An important aspect of this theory is the concept of social proof, which posits that when people are uncertain about what to do, they are more likely to mimic the actions of others they perceive as credible or comparable. Social media influencers in the modern day are similar to prominent public figures in that their posts and endorsements serve as powerful social signals for their followers [21]. One form of electronic word of mouth (eWOM) that significantly influences people’s perceptions of tourism services and destinations is the reviews, suggestions, and experiences they publish online. People often seek the opinions of others to confirm that their decisions are correct when making purchases related to tourism, which can involve risk, expense, and emotional investment [22–24]. By posting honest accounts of their travels and recommendations, influencers foster a sense of social consensus. Those who are contemplating a trip are inspired to follow suit. The exposure and perception of a brand can be changed when influential people post credible and engaging content. As a result, people’s perceptions of quality and desirability shift. To understand how digital tourism marketing leverages word of mouth, content quality, and social validation to influence client attitudes and intentions, Social Influence Theory serves as a theoretical framework.

The social influence theory explains why people listen to suggestions from influential individuals, and the source credibility theory describes how the qualities of the communicator affect the persuasiveness of such recommendations [25,26]. According to this idea, the expertise, credibility, and physical appeal of the messenger are the three factors that determine the message’s efficacy [27]. Audiences are more intended to absorb and internalize a message from an influencer they view as informed and honest. This increases consumer trust and improves consumer opinion of the advertised place or service [28,29]. The validity of the source influences the persuasiveness of the message, which in turn is affected by the medium of distribution (social media platforms) and the quality of the message itself (content attributes) [30]. Instagram, YouTube, and Facebook are digital platforms that serve as channels in influencer marketing. Social media postings and videos are seen as the message, and influencers are the ones who provide it [31,32]. A source’s appeal as a credible source aligns with the emotional connection that followers have with their influencers, which in turn enhances the acceptability of the message. Additionally, through repeated exposure and perceived authenticity, brand recognition is enhanced when trustworthy influencers continually recommend a destination. Consumer’ perceptions are shaped by these elements, which in turn influence their inclination to purchase tourism-related goods and services.

**Fig 1:**
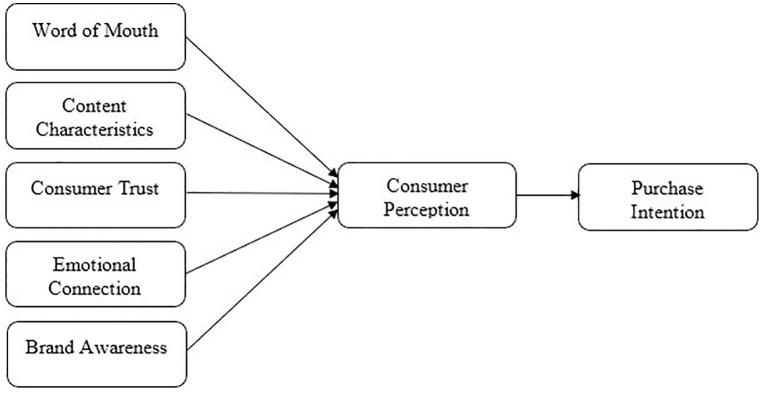
Conceptual Framework.

This paper offers a comprehensive model that describes the operation of influencer marketing in digital tourism by combining Social Influence Theory and Source Credibility Theory. The social and normative aspects of influencer impact, such as social proof and word of mouth, are explained by Social Influence Theory. In contrast, the personal and persuasive aspects, such as credibility, knowledge, and emotional resonance, are addressed by Source Credibility Theory. The cognitive (trust, content quality, brand awareness) and emotional (emotional connection) mechanisms that impact consumer perception and, by extension, buy intention are explained by these perspectives. Through this theoretical integration, a strong foundation is provided for investigating the communicative and psychological processes in Fig 1 (Conceptual framework) by which influencer marketing affects consumer behavior in the context of tourism in Bangladesh.

### 2.2 Word of mouth communication

Word-of-mouth (WOM) plays a prominent role in the tourism industry, as people usually possess extensive knowledge and feel uncertain about their decisions. Evaluating tourist services can be challenging unless one has experienced them firsthand, and specialized visitors often rely heavily on the opinions of others [33]. Influencers facilitate word of mouth by sharing experiences, reviews, and recommendations in a way that appears both authentic and genuine. Amagsila et al. [33] also confirmed the relevance of influencer word-of-mouth in forming brand perceptions, especially in travel contexts, where even if consumers did not directly experience products or services, they had confidence that the influencer’s representation of them was reliable. The potential of WOM is not limited to the tour and travel industry. Word-of-mouth recommendations from friends and family can significantly influence a person’s buying decision [34]. In the context of retail, WOM plays a significant role in influencing consumer levels of satisfaction and the extent of store patronage [35]. It increases happiness in all, and customer experience is attributed to its effectiveness, especially in the service sector [36]. The dynamics of word-of-mouth have a significant impact on its effectiveness. Emotional and persuasive messages do often add value, particularly in the restaurant business [37]. Individuals interpret issues differently depending on their trust in the messenger and the perceived depth of the information [37]. However, we would be disingenuous if we did not acknowledge that WOM has not always been a satisfactory experience. Under certain circumstances, it may generate mixed outcomes where the benefits of a particular system are gradually degraded through perceived diffuse; hence illustrating how it interacts with consumer behavior in a fugitive manner [34].

***H***_***1***_: *Word of Mouth has a positive effect on Consumer Perception.*

### 2.3 Content characteristics

It is impossible to discuss word-of-mouth (WOM) marketing without mentioning the crucial role that content plays in influencer marketing. It is the basis of all good communication and interaction. The success of an influencer is based on the content they use to convey their story, and in turn, how strongly and effectively the message resonates with their audience. Content attributes influence how people feel, behave, and engage with brands across media environments. All of which conspire to ignite a thriving culture of creativity, inspiration, and emotive connection, which bewitches, creating passion and lasting loyalty [38]. The hedonic value of content also fosters user satisfaction and intention to share this type of content, indicating that exciting information is more virally dispersed among consumers [39]. It is also worth noting that mere information provision alone may not alter attitudes, and any attractiveness of information is enhanced when it improves entertainment, enabling trustworthiness, which means consumers have a higher chance of turning into loyal customers who stay engaged with companies, which are essential elements for lasting relationships [38]. Progressive marketers understand that crafting compelling stories and sharing them in strategic locations will foster strong brand loyalty. This is especially relevant in the consumer electronics field, where the differentiator that allows firms to communicate with their customers more efficiently is quality content [40]. However, if the content is too unusual or humorous to be taken seriously, it may not be helpful to someone else. Generally, the effect of material quality is positive; however, the emotional response may be mixed, depending on the context and the expected audience reactions.

***H***_***2***_**:** *Content Characteristics have a positive effect on Consumer Perception.*

### 2.4 Consumer trust

Trust is fundamental to influencer marketing and all human relationships. It is built through credibility, dependability, and expertise [41]. Trust reduces cynicism and leads to a more positive attitude toward the destinations or services they encounter in the travel service industry. In this area, people invest significant resources and face elevated levels of risk [42]. An authentic and trustworthy influencer can increase the likelihood that consumers will choose one brand over another; however, if trust is compromised, it can be detrimental to both the influencer and the business. Trust is a pivotal factor in several domains, influencing people’s perceptions, loyalties, and purchasing behaviors. In warehousing, it dramatically impacts customer loyalty and overall perception of the firm [43]. Trust is of great importance in the insurance industry because it is closely linked to a firm’s reputation, pricing behavior, and customer loyalty [44]. In e-commerce, trust influences consumer preference by enhancing the visual appeal of online markets [45]. Studies focusing on the Go-Jek application [46] demonstrate that it has a significant impact on the perceived value and purchase intention of transport services. 1) Trust plays the role of a conduit between internet reviews, brand image, and hotel selection [47]. Furthermore, extensive evidence suggests that trust has a significant direct effect on loyalty, as believing in an organization is directly related to the level of trust that one has in it. A study of warehousing yielded a coefficient value of 0.217, indicating a significant relationship between trust and positive customer opinions [43]. Trust, as well as perceptions, has been found to affect purchase decisions based on the Go-Jek application findings significantly. Trust increases the perception of users and supports purchasing results [46].

***H***_***3***_: *Consumer Trust positively influences Consumer Perception.*

### 2.5 Emotional connection

Consumer perception has an impact on emotional connections, but emotional resonance also plays a part. Parasocial relationships, rooted in the notion of a one-sided emotional bond formed by an individual with a media persona, offer valuable insights into the intricate relationship that exists between influencers and their followers [48]. Yan et al. [49] have found that affection towards a virtual influencer leads to increased attention and interest in marketing messages. In tourism, this is particularly applicable, as dreams and emotions often drive such decision-making. Because they get to share a piece of who we are and engage as part of our story, influencers who communicate with passion and a personal/unpolished storyline can create emotional connections (that transcend beyond transactional), which makes the user engagement that much stronger. This emotional bond not only influences our perception of brands but also fosters loyalty and a lifetime of affection. When influencers share authentic travel stories of nostalgia, adventurousness, or novelty, the emotions are what people will remember and associate with the destinations they were promoting [49]. That emotional connection is the bedrock; it and its endurance simply led to a better customer experience. Evidence from Jumpa Kita Coffee Shop suggests that emotional connections with customers support the positive association, as they contribute to increased happiness accounts [50]. Emotional brand attachment has a significantly influential effect on customer attitudes and purchase intentions [51].

***H***_***4***_: *Emotional Connection has a significant positive impact on Consumer Perception.*

### 2.6 Brand awareness

Brand Awareness is probably the most important pillar that supports influencer marketing. It is the first thing that a consumer does when trying to decide. Awareness is an important predictor of brand or destination visibility to consumers, and this affects their perceptions and their intentions to purchase. Regarding the role of social media advertising in brand awareness [52], emphasize that younger people who are exposed to social media advertisements especially by influencers have a significantly higher brand awareness. The fans of tourism influencers share an ability to highlight little known destinations and experiences, thus raising its profile and exposure. Brand awareness involves more than the ability to remember or recall a brand compared to competition, but includes also: recognition of a brand when presented as a cue, some level of familiarity with the brand’s logo and marked expectations about the category in which it competes. Influencers improve brand recognition by mention particular places or services on a regular basis. This also increases the probability of people developing positive beliefs [52]. Studies have shown that a higher level of brand awareness among consumers leads to greater satisfaction with products and generates higher loyalty towards the brands. Brand awareness and perceived quality significantly foster satisfaction for Telkomsel subscriber [53]. Findings from Nineties Coffee suggests that brand awareness is positively related to customer experience and satisfaction [54]. Brand awareness affects the intentions of individuals to purchase. Several studies have shown that brand awareness, such as the brand image and perceived value of a company, significantly influences the purchase intention of customers [55]. Brand awareness itself has a critically important factor affecting consumer perceptions of brand quality as well as the value to be gained from using a product or service among matters including brand image [56]. The fact that brand awareness positively affects satisfaction, loyalty and behavioral intention is pretty much clear from the data. This indicates its importance not only in influencer marketing, but also consumer behavior.

***H***_***5***_: *Brand Awareness positively affects Consumer Perception.*

### 2.7 Consumer perception and purchase intention

There are no overnight perceptions; they evolve due to a combination of word of mouth, the quality of content being provided, trust, emotional connections, and familiarity. Chen et al. [42] argue that consumer attitudes are key to converting influencing traits into actions. Especially in tourism, where the decision-making process is intricate and involves both rational and emotional components, minimal motivational conditions are essential to facilitate a purchase. The ultimate goal of efficient influencer marketing is to encourage consumer spending. These actions do not necessarily lead to purchasing, but are considered potential leads for consumers. Studies show that when customers view influencers as reliable, appealing, and the brand as distinctive, their intentions to engage with the brand significantly increase [11,53] . Customer value, as perceived, is significant for both satisfaction and purchase intentions. Perceived value positively relates to customer satisfaction with religious travel packages, which are considered commodities, and it contributes partially to determining purchase intention [57]. Likewise, perceived value has a significant effect on online purchase intentions of trip booking applications with a t-value (7.091) [58]. SCF: Trust as a determinant of consumers’ intention to buy on the net and through e-commerce in the travel industry. High ratings and internet reviews of a company enhance consumer confidence in the firm, which in turn influences high purchase intentions [59]. The perception of trust, utility, and convenience affects customers’ attitudes and intentions towards online travel reservations in India’s National Capital Region [60]. The fit between brand image and price has a significant impact on consumers’ perceptions and buying intentions. Strong brand image and competitive price: The brand image has a positive impact on consumers’ intentions to purchase through online travel applications, with a moderate influence score of 5.253 [58]. In general, consumer attitudes are related to favorable purchase intentions; however, it is also important to recognize that consumer behavior can change over time. Reasons such as perceived risk, although they do not directly determine online trust, can affect the intention to buy.

***H***_***6***_**:** *Consumer perception positively influences purchase intention, especially in the context of travel.*

## 3. Methods

### 3.1 Research design

This study uses a quantitative, cross-sectional survey to look at how word of mouth, content characteristics, consumer trust, emotional connection, brand awareness and their impact on consumer perceptions and purchase intention in the tourism industry in Bangladesh. The research used a method called Partial Least Squares Structural Equation Modeling (PLS-SEM) to test the proposed hypotheses and look at both the measurement and structural models. We chose this method because it works well with complicated models that have more than one construct, latent variable, and interaction term, even when the data isn’t normally distributed.

### 3.2 Measurement instruments

The survey tool was made using scales from previous research (Table 1), but the wording was changed to fit the tourism context in Bangladesh. On a five-point Likert scale, 1 meant “Strongly Disagree” and 5 meant “Strongly Agree.” The breakdown of the items mentioned in Appendix A.

**Table 1:** Scale and item sources.

Constructs	Item code	Number of items	Scale (Likert)	Source
Word of Mouth	WoM	3	5 Points	Amagsila et al. [33] and [61]
Content Characteristics	CC	3	5 Points	Yao et al [62].
Consumer Trust	CT	3	5 Points	Migkos et al. [41] and Chen et al [42].
Emotional Connection	EC	3	5 Points	Yan et al [49].
Brand Awareness	BA	3	5 Points	Efendioğlu and Durmaz [52]
Consumer Perception	CP	3	5 Points	Chen et al [42].
Purchase Intention	PI	3	5 Points	Chen et al. [42] and Pourazad et al [3].

There are three items taken for each construct for this study. In SmartPLS, there needs to be at least one item (or indicator), but the ideal number varies. A minimum of three items is often recommended for a construct to have sufficient face validity and reliability, although SmartPLS can technically run with fewer items. Three technology adoption specialists meticulously reviewed the questionnaire to verify its accuracy. Subsequently, we evaluated it with 30 visitors to ascertain its clarity and reliability. Before full implementation, we made some minor adjustments to the phrasing.

### 3.3 Population and sampling

The study’s target group was people in Bangladesh who actively use social media platforms and follow travel influencers. We used a non-probability purposive sampling method to make sure that our respondents had used digital platform and familiar with influencer marketing in tourism. This made them perfect for judging usability and experience-related factors. Kline [63] said that a study should have more than 200 respondents, so we tried to get at least 300, which is enough for Structural Equation Modeling (SEM) according to [64]. From July to September 2025, we sent out a total of 500 questionnaires, both online and in person. We were left with 400 valid responses for our analysis after removing incomplete and inconsistent ones. This study employed a non-probability purposive sampling method, which is widely used in exploratory and behavioral research, particularly when the target population is large, undefined, and distributed across online environments. Since the research aimed to examine how social media influencer marketing affects tourist perceptions and purchase intentions, the most relevant respondents were active social media users who follow travel-related influencers. Reaching such respondents through random or probability-based sampling is practically infeasible due to the absence of a complete sampling frame of social media users in Bangladesh.

### 3.4 Data collection

Data has been gathered from a specific population of social media users who engage with travel influencers. While distributing the questionnaire, we enquired about each individual’s volunteer participation and meticulously reviewed the respondents’ consent, recording their opinions accordingly. The respondent’s assent explicitly indicated that the data would just be utilized for research purposes and that there would be no infringement on privacy, despite the absence of enquiries into the respondents’ personal identities. The respondent’s demographic profile is comprehensively detailed in Table 2.

**Table 2:** Demographic Profile.

Particulars		Frequency	Percentage
Gender	Male	268	67.1%
	Female	132	34.9%
Age (Years)	Below 20	26	6.5%
	21-30	360	90%
	31-40	10	2.5%
	Above 40	4	1%
Education level	Service	22	5.5%
	Business	16	4%
	Student	360	90%
	Homemaker	2	.5%
Income	Below – 20000	320	80%
	20001-30000	36	9%
	30001-40000	32	8%
	40001-60000	4	1%
	Above −60000	8	2%

Source: Survey Report, 2025.

Demographic characteristics of the survey respondents show that most are male (67.1%) and aged 21–30 years old (90%). The largest proportion is students (90%) and most of the participants earn a low income and 80% earn less than 20,000. A small percentage report that they work in the house (0.5%) and business (4%) sector. The sample is predominantly composed of younger and lower-income individuals who are all active users in social media, resembling characteristic features of influencer marketing studies implemented over tourism field.

### 3.5 Measurement model, analytical tools and technique

We assessed the measurement model using Partial Least Squares Structural Equation Modelling (PLS-SEM). The analysis was based on the reflective-formative measuring model. To analyze the data, we employed SmartPLS 4.1.1.2, a PLS-SEM software tool for advanced users. This instrument is useful for the analysis and prediction of complex relationships, in particular with a small to medium number of samples. We followed the standard two-step PLS-SEM procedure; we first addressed the measurement model and then the structural model. We further examined several critical issues in the testing of the measurement model: indicator reliability, internal consistency reliability, convergent validity, and discriminant validity. If formal theory and the appropriate sample size are unavailable, SPLS can work, but Amos does not give a proper model fit. In the words of Hair Jr et al. [65], “Both methods are complementary, not competitive.” The choice of the method originates from the goal of the research. If the existing theory needs to be tested and confirmed, CB-SEM is the chosen one. Nevertheless, for theory development and prediction purposes, PLS-SEM is better.

### 3.6 Participant’s consent

The data collection revealed a specific message regarding the goal and application of the data. The questionnaire makes clear that each respondent’s answers will be used for research goals, guaranteeing no invasion of privacy. There was a question about whether you agree to share your opinion; if you did, mark yes; if not, you do not need to fill out the questionnaire. The respondents agreed and expressed their views in response to the remark. All participants thus provided informed consent for inclusion prior to the research.

## 4. Results

A CMB test determines whether there is an effect of the data collection method on the relationships between constructs in a model [66]. This test is to check that what you have been observing are not simply artifacts of respondents or modes in the way you collected data. Table 3 displays the results of CMB, which was executed through SmartPLS version 4.1.1.5. Emotional Connection (EC) has the most deviated score of 2.392 among all components. Consumer Trust (CT) occupies the second position with 2,076 score and Brand Awareness (BA), is in the last place with a score of 1.780. All these values are orders of magnitude below the critical value of 3.3, so that common method bias is not a serious concern in this study [67]. This tells us that the dataset used for the analysis has little measurement error so we have faith in the validity and consistency of the relationships between variables. This is a confirmation that data as elicited from the respondents is reliable to be overly biased in one measurement method than the other, therefore, making it possible for us to have clearer picture of inter-coordination of several factors.

**Table 3:** Common method biased (CMB) test.

Variable	BA	CC	CP	CT	EC	PI	WoM
BA			1.780				
CC			1.314				
CP						1.000	
CT			2.076				
EC			2.392				
PI							
WoM			1.698				

Source: SmartPLS output (version 4.1.1.5).

This enables us to evaluate construct validity and reliability [64] such that our measurement model reflects the underlying conceptual constructs accurately and the observed items produce consistent measurements of these constructs. The constructs’ convergent validity, internal consistency and multicollinearity statistic results are illustrated in Table 4. The constructs are Brand Awareness (BA), Content Characteristics (CC), Consumer Perception (CP), Consumer Trust (CT), Emotional Connection (EC), Purchase Intention (PI) and Word of Mouth (WoM). The loadings of all items are above the lower limit of 0.70, from 0.782 to 0.902. It is shown that there is a strong relationship between the observed indicators and their respective latent variables. All constructs, the AVE-value varies in range 0.658 to 0.732 > .50 which is recommended s cut-off score for AVE values. This means that convergent validity is good. Internal consistency is high, from 0.740 to 0.816 (Cronbach’s alpha), and from 0.852 to 0.891 (composite reliability) above threshold values of reliability of 0.70 throughout the survey factors. The VIF for all the variables are less than 3 which suggests no multicollinearity problems across constructs [67]. Combined, the results suggest that our measurement strategy is both valid and reliable. This indicates that every single question, or indicator in the study indeed captures the expected outcome. Findings are perhaps reliable, and consistent without overlapping across variables.

**Table 4:** Construct’s validity and reliability test.

Variable	Item Code	Convergent validity		Internal consistency		Multicollinearity Statistics
		Loading > 0.70	AVE > 0.50	Cronbach’s alpha > 0.70	Composite reliability > 0.70	VIF < 3 or 5
Brand Awareness	**BA1**	0.818	0.697	0.783	0.873	1.562
	**BA2**	0.822				1.663
	**BA3**	0.864				1.682
Content Characteristics	**CC1**	0.867	0.696	0.782	0.873	1.729
	**CC2**	0.785				1.528
	**CC3**	0.848				1.664
Consumer Perception	**CP1**	0.846	0.710	0.795	0.880	1.701
	**CP2**	0.869				1.921
	**CP3**	0.811				1.573
Consumer Trust	**CT1**	0.831	0.658	0.740	0.852	1.568
	**CT2**	0.796				1.424
	**CT3**	0.806				1.450
Emotional Connection	**EC1**	0.806	0.732	0.816	0.891	1.521
	**EC2**	0.857				2.133
	**EC3**	0.902				2.402
Purchase Intention	**PI1**	0.844	0.697	0.783	0.873	1.623
	**PI2**	0.841				1.727
	**PI3**	0.819				1.564
Word of Mouth	**WoM1**	0.823	0.668	0.751	0.858	1.550
	**WoM2**	0.846				1.607
	**WoM3**	0.782				1.409

Source: SmartPLS output (version 4.1.1.5).

It is important to investigate the discriminant validity of constructs that each construct in a hypothesized model is unique and measures distinct concept than other [68]. This guarantees that the constructs are kept unique and that each variable adds info to a different part of the theoretical model. Table 5 reports the results of the discriminant validity test in terms of Heterotrait-Monotrait (HTMT) ratio and Fornell-Larcker Criterion. The uppermost HTMT objective (0.880) was achieved between Emotional Connection (EC) and Consumer Trust (CT), which reflects the high reliability of the research. All HTMT values are also below [69] the recommended cutoff point of 0.90. As for the [17], it is observed that the square root of the AVE of each construct (on the diagonal) should be greater than all correlation values between this particular and another measurement model’s constructs (off-diagonal). The square root of AVE for brand awareness (0.835) is higher than its correlations with the other constructs. The value of metric calculations for content characteristics (0.834), consumer perception (0.842), consumer trust (0.811), emotional connection (0.856), purchase intention (0.835) and Word-of-Mouth (0.817) was also high. The combined data also support strong discriminant validity of the measurement model, such that each dimension is distinct and empirically distinguishable from all other dimensions. Respondents show quite clear distinguishing the concepts used: among awareness, trust and buy intention. This suggests that the results are based on different psychological or behavioral mechanisms, rather than independent constructs.

**Table 5:** Discriminant validity test.

HTMT	Variable	BA	CC	CP	CT	EC	PI	WoM
**BA**							
**CC**	0.462						
**CP**	0.776	0.613					
**CT**	0.730	0.532	0.825				
**EC**	0.740	0.554	0.809	0.880			
**PI**	0.679	0.519	0.708	0.816	0.752		
**WoM**	0.701	0.494	0.826	0.682	0.737	0.713	
Fornell Larcker-Criterion	**Variable**	**BA**	**CC**	**CP**	**CT**	**EC**	**PI**	**WoM**
	**BA**	0.835						
	**CC**	0.368	0.834					
	**CP**	0.616	0.489	0.842				
	**CT**	0.557	0.407	0.634	0.811			
	**EC**	0.594	0.443	0.654	0.686	0.856		
	**PI**	0.537	0.406	0.561	0.622	0.605	0.835	
	**WoM**	0.535	0.386	0.639	0.509	0.579	0.546	0.817

Source: SmartPLS output (version 4.1.1.5).

Table 6 provides the results of a model fitness test using various indices, such as SRMR, d_ULS, d_G, Chi-square, and NFI, for two models: the Saturated model and the Estimated model. Model fitness tests are conducted to assess how well the proposed model fits the data compared to the saturated model, which assumes perfect fit. The SRMR value indicates the standardized root mean square residual, with a smaller value suggesting a better fit; here, the Saturated model shows an SRMR of 0.062, while the Estimated model has a slightly higher SRMR of 0.088, suggesting a slight decrease in model fit. The d_ULS and d_G indices assess the discrepancy between the model and the data using different methods, with values closer to 0 indicating better fit. The d_ULS index for the Saturated model is 0.889, whereas it is significantly higher for the Estimated model at 1.808. Similarly, the d_G value increases from 0.430 in the Saturated model to 0.498 in the Estimated model, indicating a slight degradation in model fit. The Chi-square statistic measures the overall model fit, with a smaller value representing a better fit. In this case, the Saturated model has a Chi-square of 1063.563, compared to 1161.377 for the Estimated model, again showing a reduction in fit. The NFI (Normed Fit Index) indicates the proportion of variance explained by the model, with a value closer to 1 indicating better fit. The Saturated model’s NFI value is 0.849, while the Estimated model shows a slightly lower value of 0.826, but within the acceptable range.

**Table 6:** Model fitness test.

Fitness Indices	Saturated model	Estimated model
SRMR	0.062	0.088
d_ULS	0.889	1.808
d_G	0.430	0.498
Chi-square	1063.563	1161.377
NFI	0.849	0.826

Source: SmartPLS output (version 4.1.1.5).

The R-squared and Adjusted R-squared values for the primary constructs in the model are provided for CP (Consumer Perception) and PI (Purchase Intent) in the Table 7. For CP, the R-squared value is 0.611, and the Adjusted R-squared is 0.606, indicating that approximately 61.1% of the variance in consumer perception is explained by the model, which demonstrates a strong model fit. For PI, the R-squared value is 0.315, with an Adjusted R-squared of 0.313, meaning that about 31.5% of the variance in purchase intent is explained by the model, suggesting a moderate model fit. Generally, R-squared values above 0.10 are considered to represent a moderate fit, and values above 0.25 indicate a relatively good fit [65]. These results suggest that the model provides a reasonable explanation for the variance in CP and PI, with CP showing a stronger fit than PI. While the model fits well for CP, the results for PI may require further refinement and development to enhance its explanatory power.

**Table 7:** R-square test.

Variable	R-square	R-square adjusted
CP	0.611	0.606
PI	0.315	0.313

Source: SmartPLS output (version 4.1.1.5).

The f-square values presented in the Table 8 measure the effect size of the predictors on the endogenous variables in the structural model. The f-square statistic is used to assess the strength of the relationship between each predictor and its corresponding outcome variable. According to Cohen (2013), the f-square values can be classified as small (0.02), medium (0.15), or large (0.35). In this analysis, the correlation between CP (Consumer Perception) and PI (Purchase Intent) shows an f-square value of 0.460, which is classified as a large effect, suggesting that CP has a strong influence on PI. Other relationships, such as those between BA (Brand Awareness), CC (Content Characteristics), CT (Consumer Trust), and EC (Experience Convenience), show smaller values ranging from 0.031 to 0.058, indicating weak to moderate effects on the outcome variables. These findings imply that certain variables, particularly CP, have a more substantial effect on purchase intent, while others, like brand awareness and content characteristics, exhibit relatively weaker influences on the dependent variables. The overall pattern suggests that different predictors contribute differently to the model’s outcome, with some factors having a stronger impact on consumer behavior than others.

**Table 8:** f-square test.

Variable	BA	CC	CP	CT	EC	PI	WoM
BA			0.058				
CC			0.045				
CP						0.460	
CT			0.053				
EC			0.031				
PI							
WoM			0.110				

Source: SmartPLS output (version 4.1.1.5).

**Fig 2:**
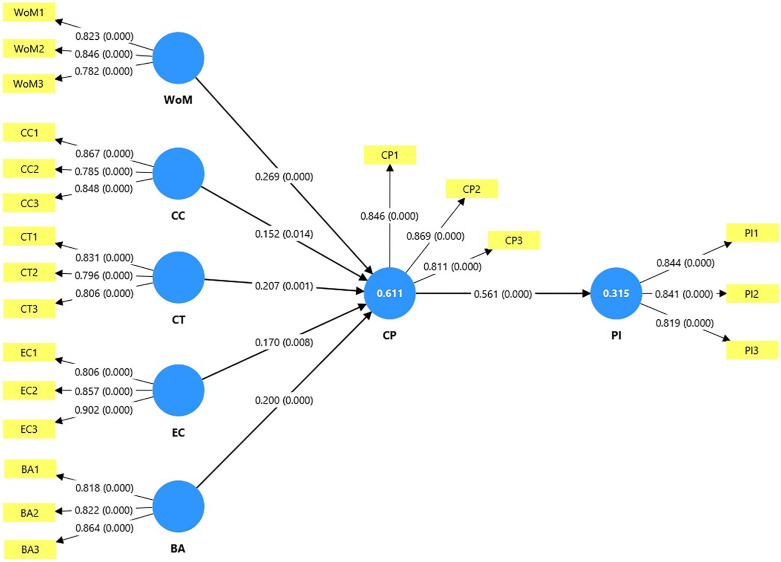
Model resolution by PLS bootstrapping. The statistical significance of the p-value with all six hypotheses (H_1_ through H_6_) is accepted, H_1_ (WoM → CP) shows strong positive relationship with value of 0.269 as well as relatively high value of p value (p < 0.001) signifying word of mouth communication is significantly affecting the consumer perception. The constructs content characteristics (β = 0.152, p < 0.05), consumer trust (β = 0.207, p < 0.05), emotional connection (β = 0.170, p < 0.05), brand awareness (β = 0.200, p < 0.001) are characterized with positive significant effects. What is more, Hypothesis H6 that links consumer perception to purchase intent presents a strong and significant effect (β = 0.561, p < 0.001). This demonstrates the role of consumer perception in shaping purchase intent. The results strongly support the proposed model in that all hypothesized relationships are significant and have a substantial effect on the dependent variables.

The path analysis in Table 9 and Fig 2 shows that six out of the six proposed hypotheses are significant at the p < 0.05 or p < 0.001 level.

**Table 9:** Hypothesis test.

Path Direction		Estimates (β)	SD	t (|β/SD|)	p values	Decision
H_1_	**WoM → CP**	0.269	0.059	4.573	0.000^b^	Accepted
H_2_	**CC → CP**	0.152	0.062	2.459	0.014^a^	Accepted
H_3_	**CT → CP**	0.207	0.063	3.269	0.001^a^	Accepted
H_4_	**EC → CP**	0.170	0.064	2.650	0.008^a^	Accepted
H_5_	**BA → CP**	0.200	0.053	3.806	0.000^b^	Accepted
H_6_	**CP → PI**	0.561	0.046	12.308	0.000^b^	Accepted

Source: SmartPLS output (version 4.1.1.5); Note: a = P < 0.05; b = P < 0.001.

### 4.1 Discussion

The findings of this study have significant consequences for influencer marketing in the travel and tourism industry. More importantly, they highlight the substantial impact of word-of-mouth perception, other content-related aspects, and consumer trust, as well as emotional connection and brand familiarity, on consumer attitudes towards it and purchase intentions, particularly in the context of trip photos displayed on social network sites (SNSs). This confirms earlier research, which shows that influencers can communicate effectively with their followers about their decisions and brand perceptions [1,33]. The Elaboration Likelihood Model [70]  suggests that credible individuals can have a significant influence on thoughts and behaviors. The identity of the influencers and the quality relevance of their content are likely vital to retaining audience engagement, as well as shaping consumer attitudes [62]. In addition, the trust of consumer, which is based on a perceived image of honesty and credibility of an influencer, is necessary to turn favorable attitudes into actual purchasing behavior [41]. The study suggested that including both positive and negative affective valences had little impact on the impression of consumers in this case. This can be explained by the rational corridor in tourism, where trust and knowledge dominate over emotions [49]. Brand familiarity was one of the significant factors that influenced consumers’ perceptions of a product. More exposure eventually leads to greater interest in the product and an increased probability of purchase [52]. This is consistent with the notion that influence outlet communication plays a significant role in consumer behavior [33,62]. Although previous analysis showed emotional connection as non-significant, the reanalysis (β = 0.170, p < 0.05) now reveals a meaningful positive relationship. This suggests that emotional bonding between followers and influencers, often developed through parasocial interactions, enhances perception of travel experiences and recommendations. In collectivist societies like Bangladesh, emotions tend to operate through relational trust rather than purely affective attachment. Thus, emotional connection may influence perception indirectly by reinforcing feelings of authenticity and identification with the influencer [71];[72]. Brand awareness also exhibited a significant effect on consumer perception (β = 0.200, p < 0.001). This finding demonstrates that influencers contribute not only to immediate behavioral responses but also to long-term brand cognition. Through consistent exposure to influencer-generated content, audiences become more familiar with destination brands and are more likely to form positive associations with them. This supports the findings of [73], who noted that digital exposure and repeated brand mentions increase recognition and trust among social media audiences. It is also found that the path coefficient of CP (β = 0.569, p < 0.001) on PI is high and positive, which means that consumers’ perception of travel influencer has a direct impact on their travel purchase [42], which emphasizes credibility and content quality as influencing how consumers perceive messages and preconditions for attitude change. The results highlight the extent to which influencer marketing influences consumer decisions regarding tourism, particularly in terms of trust [1].

This study applies the Source-Message-Channel (SMC) paradigm, Social Influence Theory, and Source Credibility Theory to the digital realm to understand how social proof, message quality, and source trustworthiness interact to influence consumer behaviour. By bringing together different ideas, we can gain a better understanding of how influencer communication in tourism marketing serves as a social and informational mechanism. Bangladesh is an emerging market to which the study applies these theoretical frameworks. The country is witnessing a surge in digital tourism marketing, and social media interactions have a profound impact on consumer decisions. Customers in Bangladesh are more receptive to influencers that provide a combination of factual information, reliable advice, and aesthetically appealing content, according to the study’s findings. There is a significant gap in the existing literature, which this study fills while also providing culturally relevant findings from a developing economy, consistent with research conducted worldwide. By demonstrating that influencer-driven traits influence tourists’ perspectives and intentions, the study successfully achieved its aims. The results showed that influencer marketing is an effective tool for promoting tourist spots. By highlighting the importance of trust, emotional resonance, and content quality in encouraging consumer engagement and influencing purchase decisions within digital tourism ecosystems, these insights enhance both theoretical frameworks and practical applications.

### 4.2 Theoretical contribution

This study enhances theoretical frameworks in several ways. First, it expands the use of the SMC (Source-Message-Channel) framework, Social Influence Theory, and Source Credibility Theory in the field of influencer marketing for tourist destinations. Despite the widespread use of these ideas in the field of advertising and communication research, a lack of comprehensive applications remains, which hinders understanding of how digital influencers affect visitor perception and purchase intentions, particularly in developing nations. This study synthesizes various theoretical frameworks to demonstrate how a combination of social influence (through word of mouth), source credibility (via trust), and message quality (through content features) affects consumer perception and future behavior. Second, by combining the rational and emotional components of consumer behavior, this study develops a thorough and scientifically supported model. Previous studies have primarily examined these aspects separately; however, the new paradigm reveals that cognitive factors (such as trust, content quality, and brand awareness) and emotional factors (such as emotional connection) interact to influence perception and, by extension, buying intention. This all-encompassing perspective deepens our theoretical understanding of how digital tourism consumers’ emotional investment and rational evaluation influence their purchasing decisions. Third, the study supports current influencer marketing models centered on the West by providing cross-cultural evidence from experimental testing of this method in Bangladesh. The results provide new theoretical insights into the impact of cultural and contextual factors on influencer-follower interactions, showing that trust and content quality are more important than emotional connection in collectivist and emerging market situations. Lastly, the study contributes to digital marketing theory by strengthening the conceptual link between social psychology and marketing communication theories through its conceptualization of influencer communication as a blend of social persuasion and brand signaling. Future research on the effectiveness of influencers across various industries and cultural contexts can build upon this comprehensive paradigm.

### 4.3 Managerial implication

Legislators, destination managers, and tourism marketers can all benefit from the research’s many suggestions for improving influencer marketing campaigns. First, marketers should prioritize collaborating with genuine and trustworthy influencers, rather than merely those with a large following, since trust and word of mouth are the most significant measures of consumers’ perceptions. More trust and reliability can be generated when influencers are genuine, share personal experiences, and engage with their audience on a personal level. Instead of relying on one-time promotional messaging, tourism companies should adopt a relationship-based influencer strategy. This approach prioritizes long-term connections that build reputation over short-term campaigns. Second, the significance of both the visual appeal and the instructional value of materials is highlighted by the fact that both content quality and originality are crucial. Suppose marketers want their influencers to showcase the destinations they visit. In that case, they should encourage them to use interactive media, such as live videos, travel vlogs, and immersive reels, along with compelling storytelling and stunning visuals. Investing in content created in collaboration with influencers and tourism boards may ensure consistency and foster an emotional connection.

Ultimately, it is crucial to effectively integrate influencer marketing with destination branding initiatives, as brand awareness has a profoundly positive impact. Businesses catering to tourists on a national or even regional scale can benefit from the consistent exposure that influencer tales provide. To increase impact, marketers can run ads across multiple platforms, including Instagram, YouTube, and TikTok. This ensures that the message is consistently seen and heard across all digital touchpoints. Fourth, influencers should be more personable in their communication since consumers’ perceptions are influenced by emotional connections. It is the responsibility of marketers to help influencers share authentic feelings, stories, and cultural experiences. Their parasocial connections and the empathy they inspire will flourish as a result. Within the realm of tourism, this type of narrative can evoke a sense of belonging and encourage visitors to visit particular locations. As a fifth point, marketers should strive to enhance the entire customer journey, beginning with the moment a consumer sees the product online and ending with the time they book it, because the client’s emotional investment in the product greatly influences the sale. Ultimately, the statistics indicate that Bangladeshi lawmakers and tourism officials should formalize influencer collaboration frameworks. To enhance the credibility and effectiveness of influencer marketing on a national level, it is crucial to establish clear ethical guidelines, promote the use of data to select influencers, and provide training on how to represent tourism responsibly. Overarchingly, these managerial decisions demonstrate that influencer marketing in the tourism industry is best viewed as an approach to communication built on trust, authenticity, and ongoing branding, rather than a means to promote anything in the near term.

## 5. Conclusion

The purpose of this research was to investigate the impact of influencer marketing on the opinions and decisions of potential customers in the Bangladeshi tourism industry. A comprehensive model that integrates both cognitive and affective aspects influencing consumer perception was developed and tested in the study, utilizing Social Influence Theory, Source Credibility Theory, and the Source-Message-Channel framework. According to the PLS-SEM study, several positive factors influence consumer perception, which is a strong predictor of purchase intention. These factors include word of mouth, content quality, trust, emotional connection, and brand awareness. By providing travelers with credible, emotionally engaging, and aesthetically appealing content, influencers have a significant impact on their feelings and travel plans. The most influential factors on perception were credibility, recommendations from others, and the quality of the information itself. As a result, being genuine and trustworthy is essential for both influencers and their followers. These advantages are amplified by emotional investment and brand recognition, demonstrating how relational and cognitive factors interact to impact digital tourism purchasing decisions. By integrating various theories of communication and behavior into a unified model, this study theoretically enhances our understanding of influencer marketing. It sheds light on its dual role as both a social persuasion process and a method for brand signaling. By applying this model to a developing economy (Bangladesh), it adds to the existing research. It provides cross-cultural evidence that improves our understanding of digital consumer behavior on a global scale. Ultimately, this study offers both theoretical and practical insights by demonstrating how influencer marketing is a powerful tool for influencing people’s minds and increasing their purchasing desire in the travel industry. The research shows that for new tourist businesses to achieve long-term success with digital marketing, a genuine connection, reliable influencers, and content that resonates emotionally with people are crucial.

### 5.1 Limitation and future work

Although this study offers valuable insights into the impact of social media influencers on tourists’ perceptions and purchase intentions, several limitations should be acknowledged. First, the research employed a non-probability purposive sampling method, which may introduce bias and limit the generalizability of the findings. The sample primarily consisted of students (90%), a group that represents a digitally active and socially engaged demographic but may not fully capture the perspectives of other consumer segments, such as working professionals, families, or international travelers. This limitation arose from the exploratory nature of the study and the absence of a comprehensive sampling frame for social media users in Bangladesh, which made probability-based methods infeasible.

Future research should therefore consider employing probability-based approaches, such as stratified or multi-stage sampling, to ensure a more representative distribution of demographic and behavioral characteristics across different consumer groups. Stratified sampling, based on factors such as age, income, education, or travel frequency, would enhance the external validity of the findings and allow for comparative analysis between distinct audience segments. Additionally, the study was limited to only Bangladesh and thus, results obtained may not be generalized across countries with different tourism role and influencer marketing situation. It would be interesting for future research to compare the effects of influencer marketing on consumers from different countries or cultures, statistically checking if they perceive it differently and if so, act in a slightly different way.

## Supporting information

S1 Data DatasetV1. (CSV)

S1 Appendix Appendix – A: Contracts and items. (DOCX)

S1 Checklist PLOSOne Human Subjects Research Checklist. (DOCX)
