# Supplementary material for: The impact of influencer marketing in the tourism industry: A digital marketing perspective
Source: PLoS One. 2025 Dec 12;20(12):e0338423. doi: 10.1371/journal.pone.0338423 (PMC12700370; doi:10.1371/journal.pone.0338423)
Supplement: S1 Appendix — (DOCX) [file pone.0338423.s002.docx]

**Appendix – A: Contracts and items**

| **Variables** | **Item Code** | **Item/Construct** |
| --- | --- | --- |
| **Word of Mouth** | WoM1 | I consider influencers’ opinions when planning my travel. |
|  | WoM2 | Reviews from influencers help me discover new travel destinations. |
|  | WoM3 | I am more interested in a destination if someone I know travels there based on an influencer’s recommendation. |
| Content characteristics | CC1 | Influencers’ content is informative and detailed. |
|  | CC2 | Visual content (photos/videos) from influencers gives me a realistic idea about the destination. |
|  | CC3 | The presentation style of influencers is engaging. |
| Consumer Trust | CT1 | I believe that the destinations or tourism services reviewed by influencers are genuine and trustworthy. |
|  | CT2 | Recommendations from influencers reduce my chances of being misled. |
|  | CT3 | I trust that influencers share their real experiences |
| Emotional Connection | EC1 | I feel emotionally connected with some influencers’ content. |
|  | EC2 | Influencers’ travel experiences inspire my own travel dreams. |
|  | EC3 | I enjoy and feel motivated by influencers’ content. |
| Brand Awareness | BA1 | I have come to know about many travel brands or agencies for the first time through influencers. |
|  | BA2 | Influencers’ content has helped me learn detailed information about tourism brands. |
|  | BA3 | Influencers play a key role in shaping my perception of travel brands. |
| Consumer Perception | CP1 | Influencer marketing enhances my perception of tourism brands. |
|  | CP2 | I have developed a positive attitude towards a destination or brand because of influencers. |
|  | CP3 | Influencers' content helps me make informed travel decisions |
| Purchase Intention | PI1 | I am likely to book a trip or service that an influencer recommends in the tourism industry. |
|  | PI2 | Content shared by travel influencers increases my intention to purchase tourism-related services. |
|  | PI3 | If a travel influencer promotes a destination, I would consider spending money to visit it. |
